# Complete genome sequence of *Vibrio parahaemolyticus* strain PH1273, isolated from aquacultured shrimp in the Philippines

**DOI:** 10.1128/MRA.00532-23

**Published:** 2023-10-19

**Authors:** John Raymond B. Salazar, Mary Nia M. Santos, Jeremy L. Palaad, Hsi-Ching Yen, Jose Louis A. Castellano, Leobert D. dela Peña, Edgar C. Amar, Erh-Min Lai, Chih-Horng Kuo

**Affiliations:** 1 The Graduate School, University of Santo Tomas, España Boulevard, Manila, Philippines; 2 Aquaculture Research and Development Division, National Fisheries Research and Development Institute, Quezon City, Philippines; 3 Institute of Plant and Microbial Biology, Academia Sinica, Taipei, Taiwan; 4 Aquaculture Department, Southeast Asian Fisheries Development Center, Tigbauan, Iloilo, Philippines; Department of Biological Sciences, Wellesley College, Wellesley, Massachusetts, USA

**Keywords:** *Vibrio*, aquaculture, shrimp, genome, *Vibrio parahaemolyticus*

## Abstract

We announce the complete genome sequence of *Vibrio parahaemolyticus* strain PH1273. This strain was collected from a *Penaeus vannamei* pond in the Philippines in 2015. Genome analysis revealed that it lacks the gene *pirAB* responsible for causing acute hepatopancreatic necrosis disease but encode multiple secretion systems and the associated effectors.

## ANNOUNCEMENT

*Vibrio parahaemolyticus* is frequently detected in marine environments and known to cause disease in penaeid shrimps (1). Consumption of seafood contaminated with this pathogen can result in severe gastrointestinal sickness (2, 3), and it represents a significant threat to the aquaculture industry (4, 5). In 2015, an outbreak of acute hepatopancreatic necrosis disease (AHPND) hampered the shrimp production in Bacolod City, Negros Occidental, Philippines (10°35′48.0″N 122°55′52.0″). One strain responsible for this outbreak, PH1339, was isolated from the hepatopancreas of a symptomatic *Penaeus vannamei* using nutrient agar (Pronadisa) with 2% NaCl and was found to harbor the plasmid-borne *pirA^vp^*/*pirB^vp^* associated with AHPND (6, 7). Another strain from the same farm, PH1273, was found to lack *pirA^vp^*/*pirB^vp^* based on PCR and was initially identified as *Vibrio coralliilyticus* based on biochemical tests (8, 9). Here, we conduct whole-genome sequencing to better understand PH1273. All kits were used according to the manufacturer’s protocols and all bioinformatics tools were used with the default settings unless stated otherwise.

A colony was picked and grown in Luria-Bertani broth with 2.5% NaCl and incubated overnight at 35°C for DNA extraction using Nanobind HMW DNA extraction kit (PacBio) for both sequencing platforms. For Illumina sequencing, genomic DNA was sheared into ~350-bp fragments using a Covaris M220 focused ultrasonicator and processed without PCR amplification using the KAPA LTP Library Preparation Kit (KK8232, Roche). Paired-end sequencing was performed on a NovaSeq 6000 using a S4 Reagent Kit v1.5 (300 cycles). A total of 23,950,752 reads were generated, producing 3,616,563,552 bp. The Illumina reads were quality-checked using FastQC v.0.11.0 (10) and trimmed using Trimmomatic v.0.36.6 (11).

For Oxford Nanopore Technologies (ONT) sequencing, DNA was further purified using AMPure XP Reagent (A63881, Beckman Coulter) and enriched for high molecular weight using the SRE XL kit (SKU 102-208-400, PacBio). The library was constructed using the Ligation Sequencing Kit (SQK-LSK109) and Native Barcoding Expansion 96 (EXP-NBD196), followed by sequencing on a MinION with an R9.4.1 flow cell (FLO-MIN106D). Basecalling was performed using Guppy v.6.4.2 with the high-accuracy model. A total of 11,040 reads were generated, with a combined sum of 244,984,022 bp and an N50 value of 31,707 bp.

The hybrid *de novo* assembly was performed using Unicycler v.0.5.0 (12), followed by polishing using Pilon v.1.20.1 (13). The genome consists of two chromosomes and two plasmids, all of which are circular contigs and have a total length of 5,580,420 bp and a GC content of 44.97%. Based on QUAST v.5.2.0 (14), the Illumina and ONT reads provided 648.1- and 43.9-fold coverage, respectively. The assembly was annotated using the National Center for Biotechnology Information Prokaryotic Genome Annotation Pipeline v.6.3 (15), which identified 5,051 coding sequences, 13 rRNA genes, 132 tRNA genes, and 4 ncRNA genes. Busco v.5.4.5 (16) indicated that the assembly has a completeness score of 100%. Genes encoding types II, III, IV, and VI secretion systems were identified. The absence of *pirA^vp^*/*pirB^vp^* was confirmed. Based on FastANI v.1.1 (17), PH1273 shares 98.2% average nucleotide identity with PH1339 (ASM3006461v2) (6) and was re-classified as *V. parahaemolyticus*.

## Data Availability

This genome project has been registered at National Center for Biotechnology Information (NCBI) under BioProject accession number PRJNA910658. The raw reads have been deposited at the NCBI Sequence Read Archive under the accession numbers SRX20585509 and SRX20251470. The genome sequence has been deposited at GenBank under the accession numbers CP114186-CP114189.
